# Suboptimal Vitamin D Status in a Population-Based Study of Asian Children: Prevalence and Relation to Allergic Diseases and Atopy

**DOI:** 10.1371/journal.pone.0099105

**Published:** 2014-06-03

**Authors:** Tsung-Chieh Yao, Yu-Ling Tu, Su-Wei Chang, Hui-Ju Tsai, Po-Wen Gu, Hsian-Chen Ning, Man-Chin Hua, Sui-Ling Liao, Ming-Han Tsai, Chih-Yung Chiu, Shen-Hao Lai, Kuo-Wei Yeh, Jing-Long Huang, Conceived and designed the experiments:, Jing-Long Huang, Tsung-Chieh Yao, Wen-I Lee, Liang-Shiou Ou, Li-Chen Chen, Kuo-Wei Yeh, Yu-Ling Tu, Man-Chin Hua, Ming-Han Tsai, Sui-Ling Liao, Shen-Hao Lai

**Affiliations:** Study Coordinator; Principal Investigators; 1 Division of Allergy, Asthma, and Rheumatology, Department of Pediatrics, Chang Gung Memorial Hospital and Chang Gung University College of Medicine, Taoyuan, Taiwan; 2 Community Medicine Research Center, Chang Gung Memorial Hospital at Keelung, Keelung, Taiwan; 3 Graduate Institute of Clinical Medical Sciences, Chang Gung University College of Medicine, Taoyuan, Taiwan; 4 Clinical Informatics and Medical Statistics Research Center, Chang Gung University College of Medicine, Taoyuan, Taiwan; 5 Division of Biostatistics and Bioinformatics, Institutes of Population Health Sciences, National Health Research Institutes, Miaoli, Taiwan; 6 Department of Pediatrics, Feinberg School of Medicine, Northwestern University, Chicago, Illinois, United States of America; 7 Department of Genome Medicine, Kaohsiung University, Kaohsiung, Taiwan; 8 Department of Laboratory Medicine, Chang Gung Memorial Hospital, Taoyuan, Taiwan; 9 Department of Pediatrics, Chang Gung Memorial Hospital at Keelung, Keelung, Taiwan; 10 Division of Pediatric Pulmonology, Department of Pediatrics, Chang Gung Memorial Hospital, Taoyuan, Taiwan; Sookmyung Women's University, Republic of Korea

## Abstract

**Background:**

New evidence shows high prevalence of vitamin D deficiency in many countries and some studies suggest a possible link between vitamin D status and allergic diseases. The objectives of this study were to determine the prevalence of suboptimal vitamin D status in a population sample of Asian children and to investigate the relationship of vitamin D status with allergic diseases and atopy.

**Methods:**

Children aged 5–18 years (N = 1315) in the Prediction of Allergies in Taiwanese CHildren (PATCH) study were evaluated using questionnaires, anthropometric measurements, and serum levels of 25-hydroxyvitamin D [25(OH)D] and total and specific immunoglobulin E (IgE).

**Results:**

The mean concentration of serum 25(OH)D was 20.4 ng/mL (SD: 7.1 ng/mL). Vitamin D deficiency (defined as serum 25(OH)D<20 ng/mL) was present in 670 subjects (51.0%), while vitamin D insufficiency (defined as serum 25(OH)D<30 ng/mL) was observed in 1187 subjects (90.3%). Older age (*P*<0.001), female gender (*P*<0.001), higher body mass index (*P* = 0.001), winter and spring seasons (compared to summer; *P* both<0.001), and passive smoking (*P* = 0.011) were independently associated with low serum 25(OH)D levels. After adjusting for potential confounders, serum 25(OH)D status had no association with asthma, rhinitis, eczema, atopy, or total serum IgE (all *P*>0.05).

**Conclusions:**

Low serum 25(OH)D levels are remarkably common in this population sample of Asian children, suggesting that millions of children living in Taiwan may have suboptimal levels of vitamin D, which should be a matter of public health concern. Our results provides epidemiological evidence against the association of vitamin D status with various allergic diseases and atopy in Asian children.

## Introduction

The understanding of vitamin D in human physiology has expanded tremendously in the last years. Historically, vitamin D is well known for its role in calcium absorption and maintenance of healthy bones, and its deficiency results in rickets in children and osteomalacia in adults. Recently, a number of studies have identified previously unanticipated roles of vitamin D in the immune system, cardiovascular system, and cancer prevention [1]. There are growing data in several countries that vitamin D deficiency is a prevalent and unrecognized health problem. It has been estimated that 1 billion people worldwide may have vitamin D deficiency or insufficiency [1]. In the NHANES III, approximately 25% to 57% of American adults and adolescents are vitamin D deficient [2]. In addition, studies in Australia, India, Lebanon, Saudi Arabia, Turkey, and the United Arab Emirates, have reported that 30 to 50% of adults and children have vitamin D deficiency [3], [4], [5], [6]. However, vitamin D status in Asian children is largely underreported.

Allergic diseases are the most common chronic diseases among children. In a global survey [7], Taiwan has the highest prevalence of allergic rhinoconjunctivitis among 6–7-year-old children in the world and the prevalence has increased by 166% during a 7-year period, attracting considerable attention to the burden of childhood allergies in this country. Our recent epidemiological study [8] has revealed that 48% of children in Keelung, Taiwan are currently symptomatic for at least 1 of 3 allergic diseases (asthma, rhinitis, and eczema).

Over the past few years, some studies have reported the relationship of vitamin D with asthma [9], [10], [11], allergic rhinitis [12], atopic dermatitis [13], and immunoglobulin E (IgE) sensitization [11], [14], [15]. Most published studies on these topics were conducted in Western populations and not population-based. The objectives of this study were to determine the prevalence of suboptimal vitamin D status in a large and well-characterized population-based cohort study of Asian children in Taiwan and to investigate the relationship of vitamin D status with allergic diseases and atopy (also called allergic sensitization).

## Methods

### Study subjects

**Figure 1:**
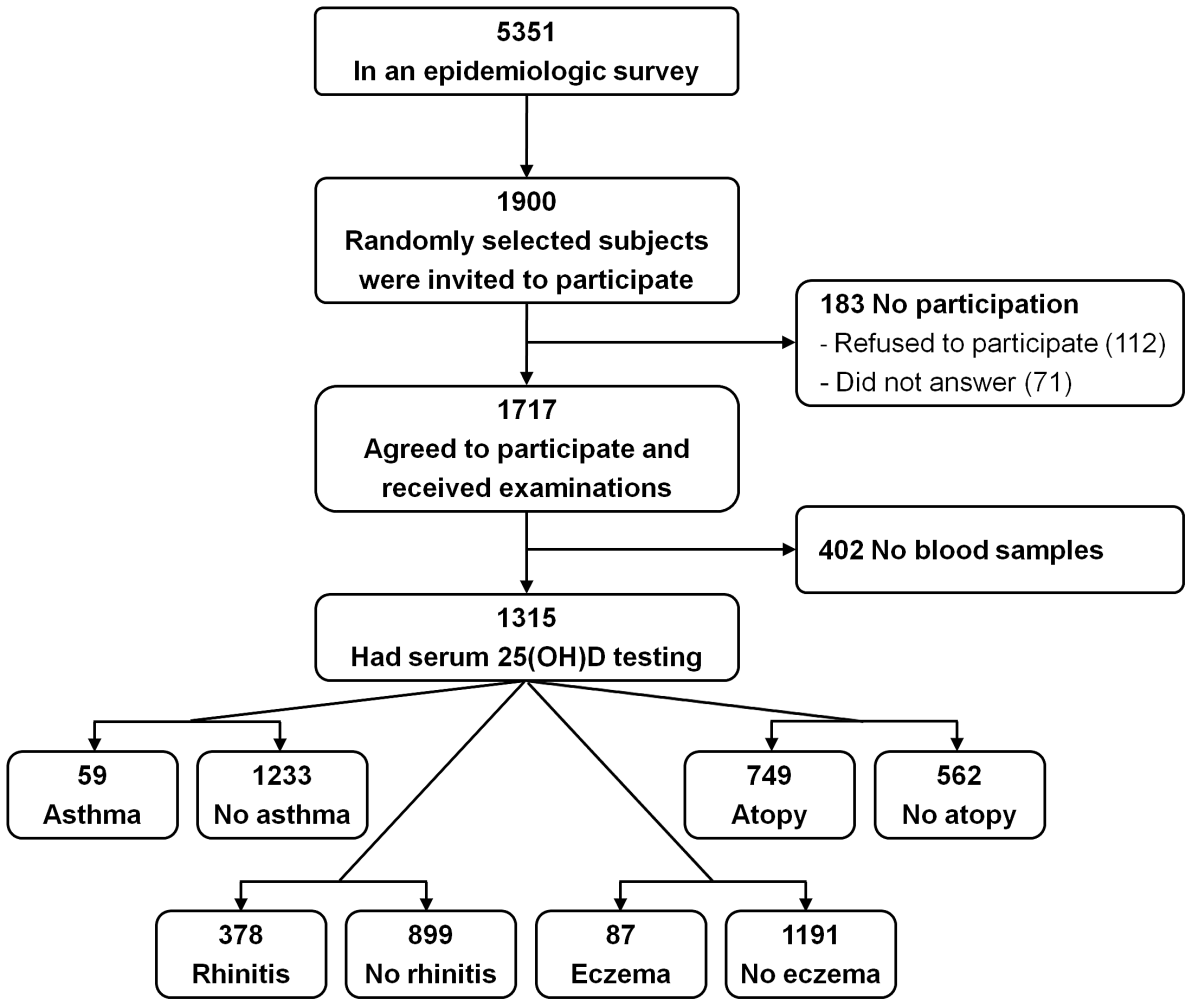
Schematic presentation of the recruitment process of the study subjects.

The Prediction of Allergies in Taiwanese CHildren (PATCH) study is a population-based prospective cohort study that was launched in 2007 in Keelung (25°N latitude), Taiwan, to investigate the epidemiology and predictive factors of asthma and allergies in children. Detailed descriptions of subject recruitment and data collection have been reported previously [8], [16], [17], [18], [19]. The subject flow diagram of the current study is presented in **Figure 1**. Briefly, study subjects were enrolled from a school-based sample of 5351 children (2616 boys, 48.9%; age, 10.4±2.9 years) in an International Study of Asthma and Allergies in Childhood (ISAAC) epidemiologic survey. Thereafter, a random sample of 1,900 subjects were invited to undergo a thorough examination and 1717 agreed to participate, representing an overall participation rate of 90.4%. Parents of all study subjects answered a questionnaire regarding demographic data, general health information, and questions related to clinical symptoms and diagnosis of allergic diseases. Serum levels of 25-hydroxyvitamin D [25(OH)D)] and allergen-specific IgE were measured in 1315 subjects whose parents agreed to blood sampling. There was no significant difference in terms of age, sex, and prevalence of allergic diseases between these 1315 subjects who provided blood samples and the original 5351 cohort members, indicating a sampling cohort representative of the general population. All subjects were born to parents who were Asian descent. The Institutional Review Board of Chang Gung Medical Foundation approved the study. The parents of all subjects provided written informed consent on behalf of their children.

### Serum 25(OH)D

Blood samples were centrifuged promptly and serum samples was stored frozen in aliquots until analysis. The stored samples were then sent to the laboratory for measurement of serum 25(OH)D at the same time. Serum 25(OH)D levels were measured by a new automated electrochemiluminescense-based assay, Elecsys Vitamin D total assay (Roche Diagnostics, Mannheim, Germany), in the central laboratory at the Chang Gung Memorial Hospital (Taoyuan, Taiwan), which is accredited by the College of American Pathologists (CAP) since 2003 with an accuracy-based 25(OH)D survey offered by the CAP being conducted semiannually. This new assay employs a competitive test principle using recombinant vitamin D binding protein allowing measurement of both 25(OH)D_2_ and 25(OH)D_3_, instead of using a monoclonal antibody against 25(OH)D_3_ in the former Elecsys Vitamin D3 assay that showed underperformance [20]. The assay is standardized against liquid-chromatography tandem mass spectrometry (LC-MS/MS) with traceability to the National Institute of Standards and Technology (NIST) standard reference material 972 [20]. In-house precision testing showed intra-assay coefficients of variation (CVs) of 3.7% and 3.4% at 15 and 32 ng/mL, respectively, and inter-assay CVs of 7.9% and 5.4% at 17 and 33 ng/mL, respectively.

### Total and allergen-specific serum immunoglobulin E

The serum level of total IgE was determined by ImmunoCAP (Phadia, Uppsala, Sweden). Specific IgE was determined by a commercial assay for IgE (ImmunoCAP Phadiatop Infant; Phadia) against the most common inhalant and food allergens (i.e. house dust mite, cat, dog, birch, timothy, ragweed, wall pellitory, egg white, cow's milk, peanut and shrimp) [21].

### Definitions of allergic diseases and atopy

Information of current and past allergic symptoms and diagnosis of allergic diseases was collected using a modified ISAAC questionnaire [22]. Asthma was defined as ever having asthma and either the occurrence of wheeze in the last 12 months or current use of asthma medications. Rhinitis and eczema were defined as ever having the two diseases, respectively, and either the presence of symptoms in the last 12 months or current use of medication for the two diseases, respectively. Atopy was defined as a positive Phadiatop Infant test result (≧0.35 PAU/L).

### Potential confounders

The following were considered potential confounding factors because of their known or plausible associations with serum 25(OH)D and outcomes: age, gender, body mass index (BMI), season of sampling, recent upper respiratory infection (URI) symptoms in the past 2 weeks, active smoking, passive smoking, and premature birth. Age and BMI were considered as continuous variables while season of sampling was categorized as spring (March to May), summer (June to August), autumn (September to November), and winter (December to February).

### Statistical analysis

All data analyses were performed using the SPSS statistical package version 15.0 for Windows (SPSS, Chicago, IL, USA). A *P*-value<0.05 was considered statistically significant. We categorized serum 25(OH)D levels as ≧30 ng/mL, between 20 and 30 ng/mL, and <20 ng/mL. Univariate analysis was performed using Student's *t*-test or ANOVA for comparing continuous variables, as appropriate, and chi-square test for comparing categorical variables. Variables with a *P*-value<0.1 in univariate analyses were included in the multivariate regression model for exploring factors associated with serum 25(OH)D. Multivariate logistic or linear regression, as appropriate, was used to examine the relationship between serum 25(OH)D status and allergic phenotypes, both unadjusted and adjusted for factors independently associated with serum 25(OH)D. We also examined serum 25(OH)D as either a dichotomous variable or a continuous variable in the analysis. Subjects with missing data on variables of interest (<3.5%) were excluded from analysis.

## Results

### Subject characteristics

The characteristics of the 1,315 subjects (645 boys; age: 10.3±2.7 years [range 5–18]) are listed in **Table 1**. The mean BMI in the study population was 18.7 kg/m^2^ (SD: 3.6). Blood samples were drawn more frequently in the spring (44.6%) and winter (33.3%), and relatively less frequently in the summer (15.7%) and autumn (6.5%). Approximately four of every ten subjects (40.6%) had recent URI symptoms in the past 2 weeks. The prevalence of active smoking was extremely low (0.7%), whereas the prevalence of passive smoking was fairly high (54.1%). A small proportion of the subjects (6.3%) were born prematurely. The prevalence of asthma, rhinitis, eczema, and atopy in the study population was 4.6%, 29.6%, 6.8%, and 57.1%, respectively.

**Table 1:** Characteristics of 1315 subjects according to serum 25(OH)D levels.

		Serum 25(OH)D level			
Characteristic	All (N = 1315)	<20 ng/mL (n = 670)	20–29.9 ng/mL (n = 517)	≧30 ng/mL (n = 128)	*P*
**Age, years (mean ± SD)**	10.3±2.7	10.9±2.6	9.7±2.5	9.5±2.8	**<0.001**
**Gender (n [%])**					
** Male**	645 (49.0)	274 (42.5)	294 (45.6)	77 (11.9)	**<0.001**
** Female**	670 (51.0)	396 (59.1)	223 (33.3)	51 (7.6)	
**BMI, kg/m^2^ (mean ± SD)**	18.7±3.6	19.1±3.8	18.4±3.4	17.6±2.9	**<0.001**
**Season (n [%])**					
** Spring (Mar to May)**	586 (44.6)	340 (58.0)	207 (35.3)	39 (6.7)	**<0.001**
** Summer (Jun to Aug)**	206 (15.7)	53 (25.7)	118 (57.3)	35 (17.0)	
** Autumn (Sep to Nov)**	85 (6.5)	28 (32.9)	48 (56.5)	9 (10.6)	
** Winter (Dec to Feb)**	438 (33.3)	249 (56.8)	144 (32.9)	45 (10.3)	
**Recent URI (n [%])**					
** Yes**	517 (40.6)	271 (52.4)	196 (37.9)	50 (9.7)	0.572
** No**	755 (59.4)	373 (49.4)	304 (40.3)	78 (10.3)	
**Active smoking (n [%])**					
** Yes**	9 (0.7)	5 (55.6)	2 (22.2)	2 (22.2)	0.339
** No**	1306 (99.3)	665 (50.9)	515 (39.4)	126 (9.6)	
**Passive smoking (n [%])**					
** Yes**	692 (54.1)	368 (53.2)	271 (39.2)	53 (7.7)	**0.005**
** No**	586 (45.9)	278 (47.4)	233 (39.8)	75 (12.8)	
**Premature birth (n [%])**					
** Yes**	80 (6.3)	35 (43.8)	39 (48.8)	6 (7.5)	0.214
** No**	1190 (93.7)	605 (50.8)	464 (39.0)	121 (10.2)	

25(OH)D, 25-hydroxyvitamin D; BMI, body mass index; URI, upper respiratory infection.

### Serum 25(OH)D levels

**Figure 2:**
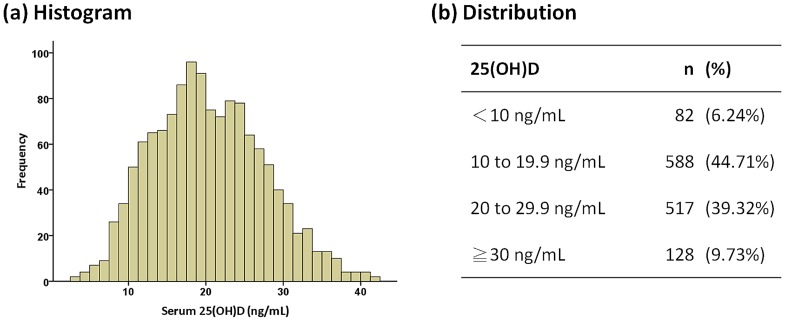
Serum 25(OH)D levels: (a) histogram, (b) distribution.

The mean concentration of serum 25(OH)D in the 1315 subjects was 20.4 ng/mL (SD: 7.1 ng/mL), with a range of 3.0 to 41.8 ng/mL. **Figure 2** showed the distribution of serum 25(OH)D levels in this population. Surprisingly, vitamin D deficiency (defined as serum 25(OH)D<20 ng/mL) was present in 670 of 1315 subjects (51.0%), while vitamin D insufficiency (defined as serum 25(OH)D<30 ng/mL) was observed in 1187 of 1315 subjects (90.3%) (**Figure 2**).

### Univariate and multivariate analyses of factors associated with serum 25(OH)D

**Figure 3:**
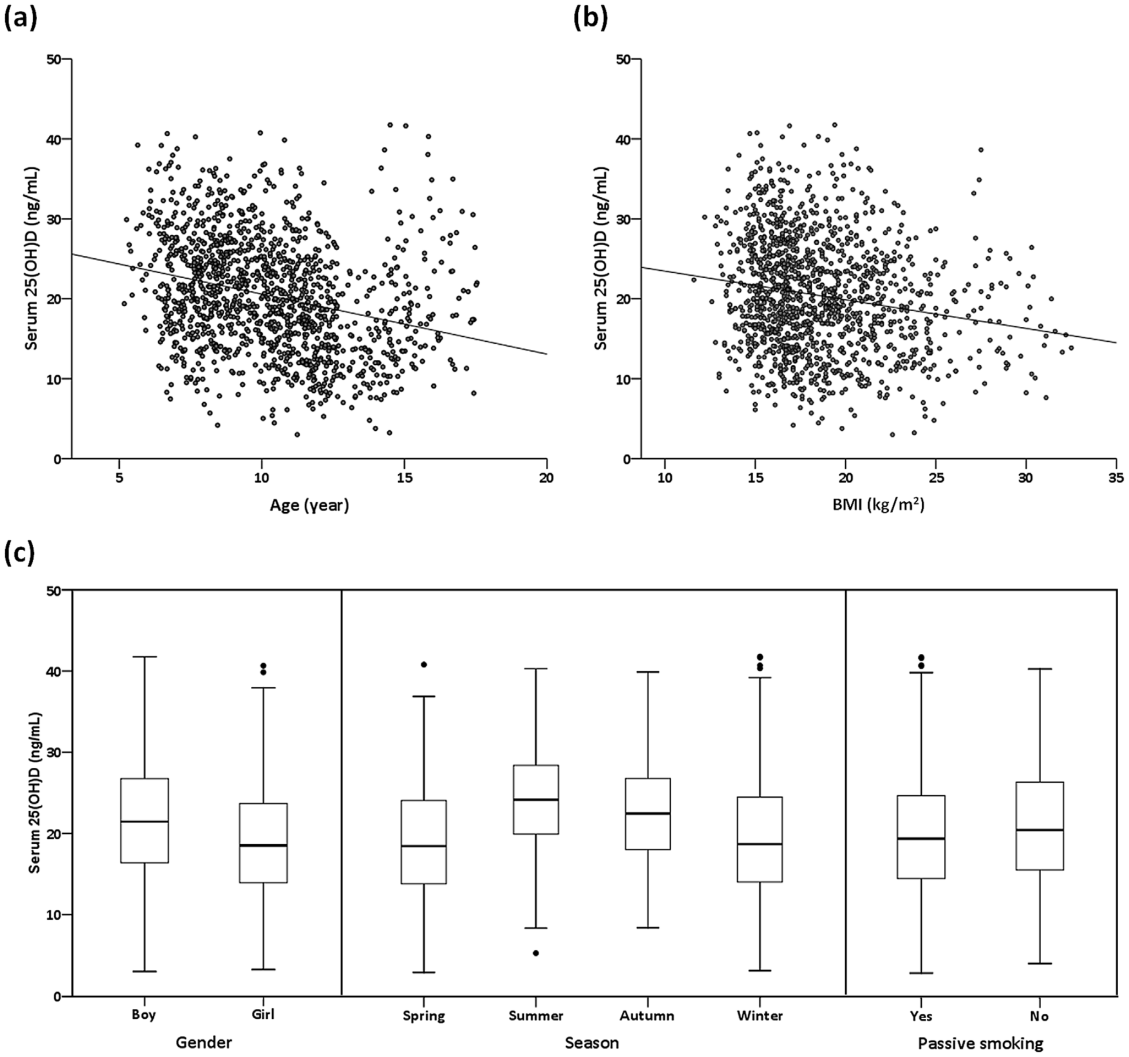
Serum 25(OH)D levels by (a) age (*r* = −0.273, *P*<0.001), (b) body mass index (*r* = −0.179, *P*<0.001), and (c) gender (*P*<0.001), season of sampling (*P*<0.001), and passive smoking (*P* = 0.003).

Univariate analysis revealed that age (*P*<0.001), gender (*P*<0.001), BMI (*P*<0.001), season (*P*<0.001), and passive smoking (*P* = 0.005) were associated with serum 25(OH)D status (**Table 1**). Serum 25(OH)D levels significantly decreased with age (*r* = −0.273, *P*<0.001; **Figure 3a**). BMI significantly and negatively correlated to serum 25(OH)D (*r* = −0.179, *P*<0.001; **Figure 3b**). Girls had significantly lower serum 25(OH)D than boys (mean ± SD, 19.2±6.9 vs. 21.6±7.2 ng/mL, *P*<0.001; **Figure 3c**). Subjects sampled in the winter (19.7±7.4 ng/mL) or spring (19.1±6.8 ng/mL) had lower serum 25(OH)D levels than did subjects sampled in the summer (24.4±6.3 ng/mL) (both *P*<0.001; **Figure 3c**). Subjects exposed to passive smoke at home had lower serum 25(OH)D levels than those did not (19.9±7.0 vs. 21.1±7.3 ng/mL, *P* = 0.003; **Figure 3c**).

Multivariate linear regression analysis of serum 25(OH)D levels was performed using variables that had a *P*-value<0.1 in univariate analyses. Older age (*P*<0.001), female gender (*P*<0.001), higher BMI (*P* = 0.001), winter and spring seasons (compared to summer; *P* both<0.001), and passive smoking (*P* = 0.011) were independently associated with low serum 25(OH)D levels (**Table 2**). Multivariate ordinal logistic regression analysis of serum 25(OH)D showed similar results, supporting the association of the aformentioned variables with serum 25(OH)D status (**Table 3**). In addition, similar results were found when we examined serum 25(OH)D as a dichotomous variable in the analysis (data not shown).

**Table 2:** Multivariate linear regression analyses for serum 25(OH)D levels.

Variable	Coefficient (95% CI)	*P*
**Age (years)**	−0.025 (−0.033∼−0.017)	**<0.001**
**Male (vs female)**	0.120 (0.080∼0.160)	**<0.001**
**BMI (kg/m^2^)**	−0.010 (−0.016∼−0.004)	**0.001**
**Season (vs summer)**		
** Spring**	−0.214 (−0.273∼−0.154)	**<0.001**
** Summer**	Reference	-
** Autumn**	−0.046 (−0.138∼0.046)	0.322
** Winter**	−0.183 (−0.245∼−0.120)	**<0.001**
**Passive smoking (vs no exposure)**	−0.051 (−0.091∼−0.012)	**0.011**

25(OH)D, 25-hydroxyvitamin D; BMI, body mass index.

**Table 3:** Multivariate ordinal logistic regression analyses for categories of serum 25(OH)D levels*.

Variable	OR (95% CI)	*P*
**Age (years)**	0.89 (0.85∼0.94)	**<0.001**
**Male (vs female)**	1.97 (1.58∼2.47)	**<0.001**
**BMI (kg/m^2^)**	0.96 (0.93∼0.99)	**0.027**
**Season (vs summer)**		
** Spring**	0.36 (0.26∼0.50)	**<0.001**
** Summer**	1.00 (Reference)	-
** Autumn**	0.79 (0.49∼1.30)	0.354
** Winter**	0.42 (0.30∼0.59)	**<0.001**
**Passive smoking (vs no exposure)**	0.76 (0.61∼0.95)	**0.016**

25(OH)D, 25-hydroxyvitamin D; OR, odds ratio; BMI, body mass index.

*Serum 25(OH)D levels were categorized into 3 ordinal categories of vitamin D status: <20, 20–29.9, and ≧30 ng/mL. The reference group for each variable is given in parentheses. The interpretation of the ORs from the ordinal logistic models is similar to the interpretation of the ORs in the binary logistic regression. For example, the OR for "male subjects (compared with female subjects)" is 1.97, which means that for male subjects, the odds of being in the ≧30 versus the <30 ng/mL category are 1.97 times the odds for female subjects, assuming that all other variables in the model are held constant. Likewise, the odds are the same for the comparison of the ≧20 versus the <20 ng/mL category (on the basis of confirmed proportional odds assumption).

### Relationship of serum 25(OH)D with allergic phenotypes

Univariate analysis demonstrated that serum 25(OH)D status was not significantly associated with asthma (*P* = 0.723), rhinitis (*P* = 0.367), eczema (*P* = 0.111), atopy (*P* = 0.171), or total IgE levels (*P* = 0.516).

Multivariate regression analysis was also undertaken to further explore the relationship of serum 25(OH)D status with allergic diseases and atopy, taking factors associated with serum 25(OH)D into consideration. After adjusting for age, gender, BMI, season of sampling, and passive smoking, serum 25(OH)D status had no association with asthma, rhinitis, eczema, atopy (**Table 4**), or total IgE levels (**Table 5**). The adjusted odds ratios for asthma seemed to increase across categories of serum 25(OH)D (1.00 [reference] for ≧30 ng/mL, 1.48 [95% CI: 0.50–4.39] for 20–29.9 ng/mL, and 1.74 [95% CI: 058–5.17] for <20 ng/mL), though not statistically significant (*P* = 0.481 and 0.322 for 20–29.9 ng/mL and <20 ng/mL, respectively) (**Table 4**).

**Table 4:** Association of serum 25(OH)D status with allergic diseases and atopy.

		Unadjusted		Adjusted**	
	Subjects n/N (%)*	OR (95% CI)	*P*	OR (95% CI)	*P*
**Asthma**					
≧30 ng/mL	4/127 (3.1)	1.00 (Reference)	-	1.00 (Reference)	-
20–29.9 ng/mL	24/509 (4.7)	1.52 (0.52∼4.47)	0.445	1.48 (0.50∼4.39)	0.481
<20 ng/mL	31/656 (4.7)	1.53 (0.53∼4.40)	0.435	1.74 (0.58∼5.17)	0.322
**Rhinitis**					
≧30 ng/mL	41/122 (33.6)	1.00 (Reference)	-	1.00 (Reference)	-
20–29.9 ng/mL	156/508 (30.7)	0.88 (0.58∼1.33)	0.535	0.93 (0.60∼1.43)	0.736
<20 ng/mL	181/647 (28.0)	0.77 (0.51∼1.16)	0.209	0.93 (0.60∼1.44)	0.737
**Eczema**					
≧30 ng/mL	9/126 (7.1)	1.00 (Reference)	-	1.00 (Reference)	-
20–29.9 ng/mL	43/504 (8.5)	1.21 (0.58∼2.56)	0.613	1.28 (0.60∼2.74)	0.522
<20 ng/mL	35/648 (5.4)	0.74 (0.35∼1.59)	0.441	0.92 (0.42∼2.04)	0.836
**Atopy**					
≧30 ng/mL	75/128 (58.6)	1.00 (Reference)	-	1.00 (Reference)	-
20–29.9 ng/mL	309/515 (60.0)	1.06 (0.72∼1.57)	0.772	1.07 (0.72∼1.60)	0.734
<20 ng/mL	365/668 (54.6)	0.85 (0.58∼1.25)	0.410	0.92 (0.62∼1.38)	0.689

25(OH)D, 25-hydroxyvitamin D; OR, odds ratio; CI, confidence interval.

*N is the sample size of each category of serum 25(OH)D and n is the number of cases in each category.

**Adjusted for age, gender, body mass index, season of sampling, and passive smoking.

**Table 5:** Association of serum 25(OH)D status with total immunoglobulin E levels.

		Unadjusted		Adjusted*	
25(OH)D (ng/mL)	Subjects	Coefficient (95% CI)	*P*	Coefficient (95% CI)	*P*
≧30 ng/mL	128	Reference	-	Reference	-
20–29.9 ng/mL	515	0.159 (−0.153∼0.471)	0.319	0.161 (−0.155∼0.476)	0.317
<20 ng/mL	668	0.076 (−0.229∼0.381)	0.624	0.116 (−0.198∼0.431)	0.468

25(OH)D, 25-hydroxyvitamin D; CI, confidence interval.

Total immunoglobulin E levels were logarithmically transformed for analysis.

*Adjusted for age, gender, body mass index, season of sampling, and passive smoking.

To further explore the association of serum 25(OH)D with allergic diseases or atopy, we also examined serum 25(OH)D as a dichotomous variable (≧30 versus the <30 ng/mL category; likewise, ≧20 versus the <20 ng/mL category) or a continuous variable in the analysis. Univariate or multivariate analysis with the serum 25(OH)D levels as a dichotomous variable or a continuous variable also did not reveal any statistically significant association of serum 25(OH)D with allergic diseases or atopy (data not shown). We also performed the above analyses stratified by gender, which did not yield any statistically significant results (data not shown).

## Discussion

New evidence shows high prevalence of vitamin D deficiency in several countries [1] and some studies suggest a possible link between vitamin D status and atopy-related phenotypes [9], [10], [11], [12], [13], [14], [15]. To our knowledge, this is the first large population-based study providing an in-depth examination of the prevalence of vitamin D deficiency and its relation to allergic phenotypes in Asian children. Main findings in the present study highlight a previously under-recognized public health issue that a substantial proportion of Asian children have suboptimal levels of vitamin D, especially those who are older in age, are female, have a higher BMI, are evaluated in winter or spring season, and are exposed to passive smoke. Another important finding is that our data does not support associations of vitamin D status with allergic diseases and atopy in this population.

In recent years, growing attention has been paid to the vitamin D status of Western populations, but there is a paucity of data in the Asia, particularly in children, on the prevalence and predictors of vitamin D deficiency. Determination of vitamin D status in children is generally more difficult in a large number of children than that in adults because sampling issues are more challenging. This cohort is perhaps one of the largest study to date that measures serum 25(OH)D in a population of 1315 Asian children over a wide range in the community. At present, the best indicator of vitamin D status is the serum 25(OH)D [1]. While there is no universally accepted consensus yet on serum 25(OH)D thresholds to define optimal vitamin D status, vitamin D deficiency is defined by most experts as <20 ng/mL, and insufficiency as <30 ng/mL [1], [23], with threshold values based on optimal bone health [24]. When these cut-offs are applied to children aged 5 to 18 years in the current study, more than half (51%) of the children have vitamin D deficiency and 9 of every 10 children have insufficient vitamin D levels. Potential explanations for the remarkably high prevalence of low vitamin D status observed in this population include, but are not limited to, skin pigmentation, the substantial number of rainy days (i.e., an average of 15.9 days with at least 0.1 mm rain per month during the study period), the widespread use of sunscreens on children, and perhaps also dietary factors. This study identifies a previously under-recognized public health issue of vitamin D deficiency in Asian children and therefore calls for careful consideration of vitamin D supplementation particularly in at-risk children to further optimize health.

Given that exposure to sunlight, specifically UVB, is believed to provide most of the vitamin D requirements of the human population [25], the seasonal variation of vitamin D levels in the current study is expected. The inverse association of vitamin D status with age during childhood and adolescence in this population is of interest, which is in accordance with previous studies [26], [27], [28], [29], [30]. A plausible explanation is that older children and adolescents may have less exposure to sunlight due to fewer incentives and opportunities for outdoor activities. Indeed, it has been shown previously that a consistent decline in physical activity is found over the school age years, with males decreasing about 2.7% per year and females decreasing about 7.4% per year [31]. Consistent with previous studies [5], [27], [28], [29], [30], [32], [33], [34], [35], gender and BMI each has an independent effect on serum vitamin D levels in the current study. It has been demonstrated that the prevalence of physical inactivity is higher in girls than boys and is higher in obese children than their normal-weight peers [31]. It is therefore possible that gender and BMI may act as surrogate indicators for sunlight exposure through their association with physical activity. In addition to less sunlight exposure due to sedentary lifestyle, the observed inverse association between BMI and vitamin D levels could be explained by other possibilities, including poor diet and sequestration of vitamin D in fat tissues [36]. The associations in our study between low vitamin D status and exposure to passive smoke, after adjusting for confounders, have not, to our knowledge, been previously reported, and the mechanisms remain unclear and warrant further study.

In the current study, allergic phenotypes including asthma, rhinitis, eczema, atopy, and total IgE levels are not associated with serum 25(OH)D, in contrast with previous studies that demonstrate relationships between various allergic phenotypes and vitamin D status [9], [10], [11], [12], [13], [14], [15]. Two case-control studies provide epidemiological support for the association between vitamin D deficiency and asthma [9], [10]. A longitudinal cohort study [11] reveals that serum vitamin D levels at age 6 years are not associated with concurrent asthma at that age but are significant predictors of subsequent asthma at age 14 years. The cross-sectional analysis in the current study shows no significant association between serum 25(OH)D and concurrent asthma, although the role of serum 25(OH)D levels as predictors of subsequent asthma remains to be defined in the longitudinal follow-up of the cohort subjects which is currently underway. Although there is also emerging evidence from recent studies suggesting the association of vitamin D status with allergic rhinitis [12], atopic dermatitis [13], and IgE sensitization [11], [14], [15], the findings from the current study do not support the previous research. Taken together, the current study provides epidemiological evidence against a strong link between vitamin D status and various allergic phenotypes.

This study has several notable strengths. The representative sampling of children across a broad age range in the community, large sample size with robust data collection, incorporation of objective markers of atopy, and thorough analysis, all add strengths to the results of this study. This study has some limitations, including its cross-sectional design and lack of data on dietary and supplemental vitamin D intake and physical activity. Some might argue that the small proportion of children with sufficient serum 25(OH)D levels (≧30 ng/mL) might have limited our ability to discern associations. In our opinion, however, the argument is very unlikely since the repeat analyses by treating serum 25(OH)D levels as either a dichotomous variable or a continuous variable do not significantly alter the results. Although it is possible that this relatively large study has not been adequately powered to detect associations of modest effect, the effect size observed in this study is hardly likely to be clinically significant even statistical significance would have been attained with a larger sample size. It will be interesting to investigate in future follow-ups of this longitudinal cohort if serum 25(OH)D levels predict the subsequent development of allergic phenotypes.

In conclusion, low serum 25(OH) D levels are remarkably common in this population-based sample of Asian children aged 5 to 18 years, suggesting that millions of children living in Taiwan may have suboptimal levels of vitamin D, which should be a matter of concern for public health authorities and professionals and also for families with children. This population-based study provides evidence against the association of vitamin D status with allergic diseases and atopy in Asian children. The relationship between vitamin D status and allergic diseases thus merits further study.
